# Increased Expression of lncRNA AC000120.7 and SENP3-EIF4A1 in Patients with Acute Respiratory Distress Syndrome Induced by SARS-CoV-2 Infection: A Pilot Study

**DOI:** 10.3390/microorganisms11092342

**Published:** 2023-09-19

**Authors:** Javier González-Ramírez, Ana Gabriela Leija-Montoya, Nicolás Serafín-Higuera, Carlos A. Guzmán-Martín, Luis M. Amezcua-Guerra, Carlos Olvera-Sandoval, Jesús René Machado-Contreras, Armando Ruiz-Hernández, Adrián Hernández-Díazcouder, Julia Dolores Estrada-Guzmán, Fausto Sánchez-Muñoz

**Affiliations:** 1 Facultad de Enfermería, Universidad Autónoma de Baja California, Av. Álvaro Obregón y Calle “G” S/N, Col. Nueva, Mexicali 21100, Baja California, Mexico; javier.gonzalez.ramirez@uabc.edu.mx; 2 Laboratorio de Biología Celular, Unidad de Ciencias de la Salud Campus Mexicali, Universidad Autónoma de Baja California, Calle de la Claridad S/N, Col. Plutarco Elías Calles, Mexicali 21376, Baja California, Mexico; 3 Facultad de Medicina Mexicali, Universidad Autónoma de Baja California, Dr. Humberto Torres Sanginés S/N, Centro Cívico, Mexicali 21000, Baja California, Mexico; gabriela.leija@uabc.edu.mx (A.G.L.-M.); olvera.carlos@uabc.edu.mx (C.O.-S.); rene.machado@uabc.edu.mx (J.R.M.-C.); armando.ruiz.hernandez@uabc.edu.mx (A.R.-H.); juliaestrada@uabc.edu.mx (J.D.E.-G.); 4 Facultad de Odontología, Universidad Autónoma de Baja California, Zotoluca S/N, Fracc. Calafia, Mexicali 21040, Baja California, Mexico; nserafin@uabc.edu.mx; 5 Departamento de Inmunología, Instituto Nacional de Cardiología Ignacio Chávez, Juan Badiano No. 1, Col. Sección XVI, Tlalpan, Mexico City 14080, Mexico; gmcarlos93@gmail.com (C.A.G.-M.); lmamezcuag@gmail.com (L.M.A.-G.); adrian.hernandez.diazc@hotmail.com (A.H.-D.); 6 Laboratorio de Investigación en Obesidad y Asma, Hospital Infantil de México Federico Gómez, Calle Doctor Márquez 162, Cuauhtémoc, Mexico City 06720, Mexico

**Keywords:** COVID-19, SARS-CoV-2, long non-coding RNA, acute respiratory distress syndrome

## Abstract

COVID-19, a disease caused by the SARS-CoV-2 virus, poses significant threats to the respiratory system and other vital organs. Long non-coding RNAs have emerged as influential epigenetic regulators and promising biomarkers in respiratory ailments. The objective of this study was to identify candidate lncRNAs in SARS-CoV-2-positive individuals compared to SARS-CoV-2-negative individuals and investigate their potential association with ARDS-CoV-2 (acute respiratory distress syndrome). Employing qRT-PCR, we meticulously examined the expression profiles of a panel comprising 84 inflammation-related lncRNAs in individuals presenting upper respiratory infection symptoms, categorizing them into those testing negative or positive for SARS-CoV-2. Notably, first-phase PSD individuals exhibited significantly elevated levels of AC000120.7 and SENP3-EIF4A1. In addition, we measured the expression of two lncRNAs, AC000120.7 and SENP3-EIF4A1, in patients with ARDS unrelated to SARS-CoV-2 (*n* = 5) and patients with ARDS induced by SARS-CoV-2 (ARDS-CoV-2, *n* = 10), and interestingly, expression was also higher among patients with ARDS. Intriguingly, our interaction pathway analysis unveiled potential interactions between lncRNA AC000120.7, various microRNAs, and genes associated with inflammation. This study found higher expression levels of lncRNAs AC000120.7 and SENP3-EIF4A1 in the context of infection-positive COVID-19, particularly within the complex landscape of ARDS.

## 1. Introduction

In December 2019, an outbreak of severe pneumonia caused by a novel coronavirus was identified, leading to the recognition of severe acute respiratory syndrome coronavirus 2 (SARS-CoV-2) as the causative agent of coronavirus disease 2019 (COVID-19) [1]. While most patients with COVID-19 experience mild symptoms, many patients may develop severe disease. Diagnostic considerations of COVID-19 include recent-onset fever and upper or lower respiratory infection symptoms [2,3]. To confirm the diagnosis, testing for SARS-CoV-2 in samples from the upper respiratory tract using PCR is essential [4]. Early onset of dyspnea and hypoxemia may lead to severe forms of COVID-19, which are associated with the development of acute respiratory distress syndrome (ARDS). These patients face a high mortality rate due to shock, thrombosis, and multiple organ dysfunction [5,6].

Long non-coding RNAs (lncRNAs) are transcripts that exceed 200 nucleotides in length and lack protein-coding capacity [7]. LncRNAs exhibit significant heterogeneity and possess remarkable functional adaptability. They can adapt to different molecular structures and establish distinct molecular interactions [8]. LncRNAs are involved in transcriptional regulation and RNA processing, functioning as “molecular decoys” for microRNAs (miRNAs), serving as “scaffolds” for proteins, and even as precursors for miRNAs [9]. These lncRNAs have emerged as promising targets for clinical and therapeutic research [10]. SARS-CoV-2 studies have shown an association between lncRNA expression patterns and the severity of infection [11]. However, the number of studies exploring this relationship remains low, highlighting a knowledge gap that needs to be addressed [12]. Therefore, the aim of this study was to identify potential candidate lncRNAs in individuals with a confirmed SARS-CoV-2 diagnosis in comparison to those who tested negative for the virus. Additionally, we sought to explore the potential associations between the expression of these lncRNAs and the development of ARDS.

## 2. Materials and Methods

### 2.1. Ethics Statement

The study protocol received approval from the Ethics Committee of the Faculty of Medicine of Mexicali, with registration number FMM/CEI-FMM/003/2021-1. Prior to their inclusion in the study, all participants provided written informed consent. For the second phase of the study, the patients were required to sign an informed consent form upon their hospital admission. This phase was conducted at the Ignacio Chávez National Institute of Cardiology, located in Mexico City, Mexico. The study was approved by the local ethics committee, under project number 20-1186. All procedures were carried out in accordance with the 2013 Declaration of Helsinki, its addenda, and local regulations.

### 2.2. Subjects

We conducted a two-phase investigation: In the initial phase, we recruited a total of six patients with upper respiratory infection symptoms from the COVID-19 Diagnosis Center of the Faculty of Medicine Mexicali UABC. Among them, three patients tested negative for SARS-CoV-2 diagnosis (NSD), and three tested positive for SARS-CoV-2 diagnosis (PSD), through RT-PCR. Inclusion criteria: The participants enrolled in this study were individuals seeking evaluation at the COVID-19 Diagnostic Center of the Mexicali School of Medicine, presenting with at least one characteristic symptom of SARS-CoV-2 infection, including but not limited to cough, fever, headache, myalgia, and arthralgia. Eligible participants fell within the age range of 20 to 60 years, with no gender restrictions. Exclusion criteria: Excluded from the study were individuals who were pregnant, asymptomatic individuals, those currently undergoing antiviral treatment, or samples that were deemed to be in suboptimal condition or poorly preserved. Elimination criteria: Samples with insufficient volume, low leukocyte recovery, evidence of RNA degradation, improper storage conditions, or sample contamination were subject to elimination from the study.

Biological specimens were collected from unvaccinated individuals. Nasopharyngeal samples were obtained using swabs, while peripheral blood samples were collected and stored in EDTA tubes. The samples were promptly refrigerated between 4 and 8 °C until use.

The second phase of the study was conducted at the Ignacio Chávez National Institute of Cardiology in Mexico City, Mexico. In this phase, we recruited a total of 15 patients from the intensive care unit, all of whom had been diagnosed with ARDS. Among these patients, five had ARDS unrelated to SARS-CoV-2 (ARDS), while the remaining ten were diagnosed with ARDS caused by SARS-CoV-2 (ARDSCoV-2). The inclusion criteria were as follows: confirmed diagnosis of COVID-19 based on a positive PCR test for SARS-CoV-2 for the ARDS-CoV-2 group and at least 2 negative PCR tests for SARS-CoV-2 for the ARDS group without COVID-19; patients over 18 years old admitted to the intensive care unit (ICU) at Ignacio Chávez National Institute of Cardiology for the treatment of severe acute respiratory distress syndrome. Exclusion criteria: if patients or their legally authorized representatives refused to provide informed consent for participation in the study.

### 2.3. SARS-CoV-2 Detection in the NSD and PSD Groups

The extraction of viral RNA in nasopharyngeal swab samples was performed using the Quick RNA MiniPrep Kit (Zymo Research, Irvine, CA, USA), following the supplier’s instructions. RT-qPCR was performed using the protocol published by the US Centers for Disease Control and Prevention (CDC), Atlanta (CDC 2019-nCoV Real-Time RT-PCR Diagnostic Panel), which detects SARS-CoV-2-specific gene fragments, and the human RNase P housekeeping gene was determined as an extraction control.

The qRT-PCR reaction was performed on a CFX96 (Bio-Rad, Hercules, CA, USA) with the SuperScript™ III One-Step RT-PCR System and Platinum™ Taq DNA Polymerase kits (Thermo Fisher, Waltham, MA, USA), along with specific primers and probes for SARS-CoV-2 as described above. The thermocycling conditions were RT at 55 °C for 10 min, inactivation at 95 °C for 2 min, denaturation at 95 °C for 5 s, amplification at 60 °C for 30 s, and the last two steps were repeated for 45 cycles.

### 2.4. Isolation of Leukocytes in the NSD and PSD Groups

Samples of blood were collected and dispensed in KEDTA tubes (Becton Dickinson, Franklin Lakes, NJ, USA). Erythrocytes from blood were lysed using an ACK (ammonium–chloride–potassium) lysing buffer. The tubes were mixed (8 inversions) and incubated for 3–5 h at 4 °C until processing. Then, 0.8 mL of whole blood was mixed with 14 mL of ACK lysing buffer at room temperature and incubated for 8 min. After centrifugation at 300× *g* for 5 min at room temperature, the supernatant was collected. The pellet was washed twice with 5 mL of phosphate-buffered saline and centrifuged at 4 °C. White blood cell samples were mixed with 1 mL of TRIzol reagent (Invitrogen, Waltham, MA, USA), followed by storage at −20 °C until processing.

### 2.5. Total RNA Isolation and cDNA Synthesis in the NSD and PSD Groups

Total RNA was extracted from leukocytes by the Tripure method, following the manufacturer’s protocol (Roche; Basel, Switzerland). The RNA obtained from each sample was quantified using a nanophotometer to ensure accurate measurements. The quality of the RNA was assessed by determining the 260/280 nm absorbance ratio for each RNA sample, which was consistently recorded as 1.9 ± 0.2. The RNA isolated from leukocytes was immediately converted to cDNA, as described below.

### 2.6. cDNA Synthesis and qRT-PCR Analysis in the NSD and PSD Groups

The synthesis of cDNA was performed using 1 μg of total RNA with an RT2 First Strand Kit (QIAGEN, Hilden, Germany). The cDNA manufacturing protocol included a treatment to avoid DNA carryover (DNase I for 30 min at 42 °C), and it was performed according to the manufacturer’s instructions. The qRT-PCR was performed using RT2 SYBR^®^ Green qPCR Master Mix (Qiagen; Hilden, Germany). The reaction (25 μL) was placed into the wells of the QIAGEN Inflammatory Responses RT2 lncRNA PCR array kit (Qiagen; Hilden, Germany), which contains the pairs of specific, predesigned, and laboratory-verified oligonucleotides. The lncRNA expression levels were normalized to those of the internal control RN7SK. Values are the means ± SE. The relative expression values were tested with an unpaired *t*-test (* *p* < 0.05).

### 2.7. Total RNA Isolation in the ARDS and ARDS-CoV-2 Groups

Total RNA was extracted from total blood by the Tripure method, following the manufacturer’s protocol (Roche; Basel, Switzerland). To remove any DNA contamination, the isolated RNA was treated with DNase (Thermo Fisher Scientific, Waltham, MA, USA). The quality and purity of the RNA were assessed by determining the 260/280 nm absorbance ratio for each RNA sample, using a nanophotometer. The isolated RNA was immediately used for RT-qPCR.

### 2.8. RT-qPCR Analysis in the ARDS and ARDS-CoV-2 Groups

LncRNAs were determined using one-step RT-qPCR with the RT 2 lncRNA qPCR assay for AC000120.7 (Qiagen catalog number: LPH15155A-200, NCBI Entrez Gene: ENST00000414227) and SENP3-EIF4A1 (Qiagen catalog number: LPH24757A-200, NCBI Entrez Gene: ENST00000579777) (Qiagen; Hilden, Germany). Each one-step RT-qPCR reaction used 90 ng of total RNA that had been isolated following the manufacturer’s protocol (Qiagen; Hilden, Germany). The one-step RT-qPCR reaction program consisted of 10 min at 50 °C for reverse transcription and 2 min at 95 °C for initial PCR activation, followed by 40 cycles at 95 °C for 5 s and 60 °C at 10 s. PCR was performed using a CFX96 system (Bio-Rad; CA, USA). The lncRNAs’ relative concentrations were normalized with Ct values of GAPDH (primer L: AGCCACATCGCTCAGACAC, primer R: GCCCAATACGACCAAATCC), and the values were calculated using the 2^−ΔCt^ formula.

### 2.9. Statistical Analysis

Statistical analyses were performed using SPSS v26.0 software (SPSS Inc., Chicago, IL, USA) for all quantitative data, while GraphPad Prism v9 software was employed for data visualization. Quantitative variables were presented as either medians with interquartile ranges (IQRs) or means with standard deviations, depending on the nature of the data, while qualitative data were expressed as frequencies and percentages. In the first phase, differences in lncRNA expression were assessed using Welch’s test. In the second phase, the Mann–Whitney U test was utilized. A *p*-value of less than 0.05 was considered statistically significant.

### 2.10. Pathway Network Interaction Analysis

**Figure 1:**
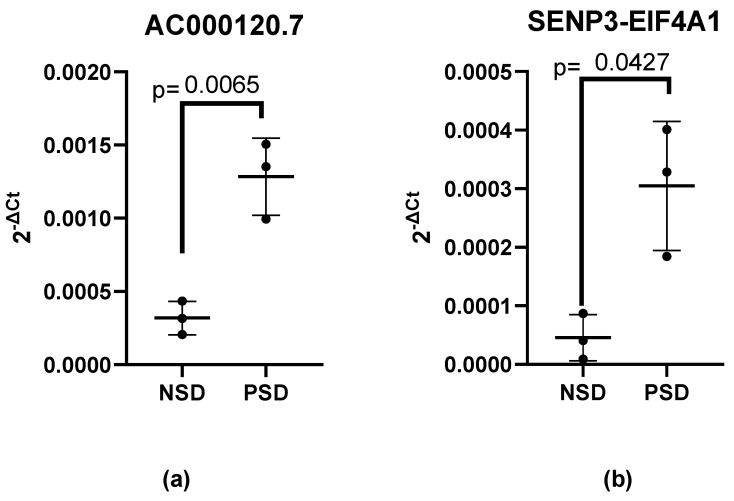
(**a**) AC000120.7 and (**b**) SENP3-EIF4A1 expression levels in leukocytes of patients: NSD (negative for SARS-CoV-2) and PSD (positive for SARS-CoV-2). The lncRNAs’ expression levels were normalized to those of the internal control RNA component of 7SK nuclear ribonucleoprotein (RN7SK). Values are the means ± SE. The relative expression values were tested with unpaired *t*-tests.

We constructed an interaction map of the lncRNA pathways, focusing on the predicted interactions of lncRNAs AC000120.7 and SENP3-EIF4A1 with various microRNAs and genes. These interactions were established through base-pairing interactions sourced from reputable databases: miRnet.ca (https://www.mirnet.ca/. Accessed on: 5 July 2023), the RNA interactome Database (https://www.rna-society.org/rnainter/, Accessed on: 5 July 2023), RNAcentral (https://rnacentral.org/ Accessed on: 5 July 2023; https://diana.e-ce.uth.gr/lncbasev3. Accessed on: 5 July 2023), LNCipedia (https://lncipedia.org/. Accessed on: 5 July 2023), and LncRRIsearch (http://rtools.cbrc.jp/LncRRIsearch/. Accessed on: 5 July 2023). Network analysis was performed by integrating all of the information with Cytoscape 3.9.1. These interactions can be seen in Figure 1a. Furthermore, we conducted enrichment analysis using the bioinformatics tool DAVID Bioinformatics Resources (https://david.ncifcrf.gov/. Accessed on: 6 July 2023) to identify and explore the implicated pathways.

## 3. Results

A two-phase pilot study was conducted; in the first phase, three NSD and three PSD patients with flu-like symptoms participated. Their characterization and main symptomatology are shown in Table 1.

In first phase, a comprehensive analysis was conducted on a set of 84 genes associated with the human inflammatory response. The results revealed elevated expression levels of lncRNA AC000120.7 (means: 0.001284 vs. 0.0003186, 95% CI: 0.0004081 to 0.001523, effect size: 4.764, actual statistical power: 0.987, *p* = 0.0130) and SENP3-EIF4A1 (means: 0.0003047 vs. 4.546 × 10^−5^, 95% CI: 1.787 × 10^−5^ to 0.0005007, effect size: 3.133, actual statistical power: 0.814, *p* = 0.0427) in patients with PSD as compared to those with NSD, respectively (Figure 1a,b).

**Figure 2:**
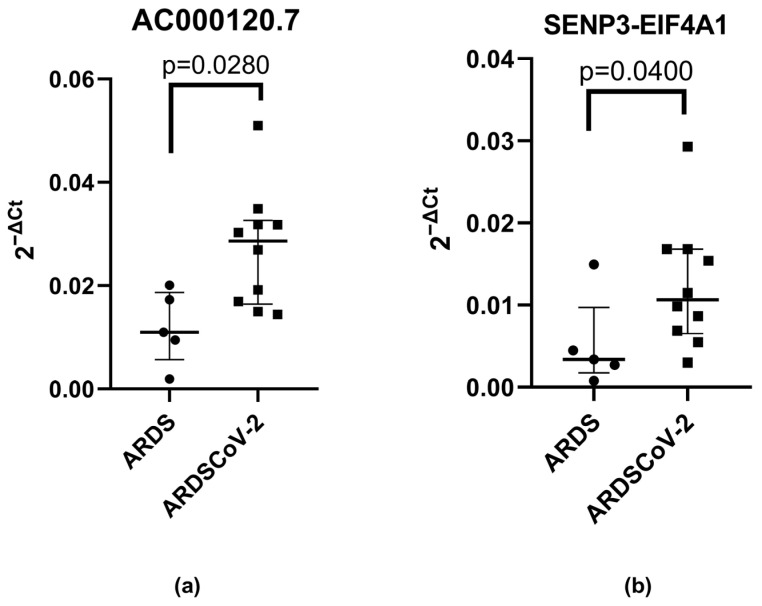
(**a**) AC000120.7 and (**b**) SENP3-EIF4A1 expression levels in the blood of patients with ARDS (acute respiratory distress syndrome not caused by SARS-CoV-2) and ARDS-CoV-2 (patients with acute respiratory distress syndrome caused by SARS-CoV-2). The relative expression values were tested with the Mann–Whitney test.

In the second phase of the study, it was observed that higher expression of AC000120.7 (median: 0.02860, 95% CI: 0.01911 to 0.03533 vs. 0.01097, 95% CI: 0.003105 to 0.02078, effect size: 1.6139, actual power: 0.9636142, *p* = 0.0280) and SENP3-EIF4A1 (median: 0.01065, 95% CI: 0.006911 to 0.01781 vs. 0.003380, 95% CI: −0.001671 to 0.01220, effect size: 1.062352, actual power: 0.9506665, *p* = 0.0400) remained in patients diagnosed with ARDS-CoV-2 compared to patients with ARDS (Figure 2a,b). The main features of the patients are presented in Table 2.

**Figure 3:**
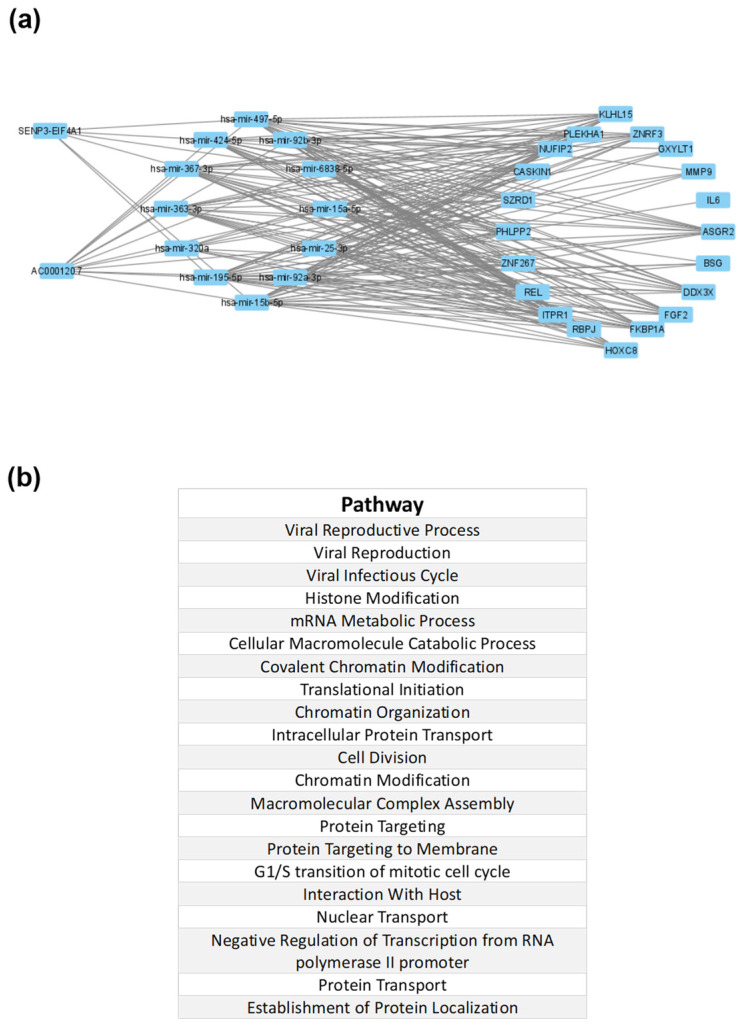
Bioinformatics analysis: (**a**) Interaction pathway with the predicted interactions of lncRNAs AC000120.7 and SENP-EIF4A1 with diverse microRNAs and genes; interestingly, we can appreciate some inflammation-related COVID-19 genes previously described by other authors, such as IL-6, basigin, and MMP9. (**b**) Enrichment analysis of the interaction network, in which several viral pathways are implicated.

Finally, utilizing the bioinformatics tool miRNet 2.0 (available at https://www.mirnet.ca/. Accessed on: 5 July 2023), we constructed an interaction pathway that unveiled predicted interactions involving lncRNA AC000120.7 (also called KRIT1) with various miRNAs and genes. Notably, within this pathway, we observed the presence of several genes associated with inflammation and COVID-19 that have been previously identified by other researchers, including IL-6, basigin, and MMP9 (Figure 3).

## 4. Discussion

In this pilot study, our aim was to assess the expression levels of inflammation-related lncRNAs in leukocyte samples obtained from both SARS-CoV-2-positive and SARS-CoV-2-negative patients, especially in those with acute respiratory distress syndrome, through qRT-PCR. Interestingly, we observed elevated expression levels of lncRNAs AC000120.7 and SENP3-EIF4A1 in PSD patients compared to NSD patients. Likewise, we observed that association with SARS-CoV-2 infection persisted in the subsequent study involving patients with ARDS. Additionally, a bioinformatics analysis identified the interaction of these lncRNAs with various miRNAs and genes, including inflammation- and COVID-19-associated genes like IL-6, basigin, and MMP9.

To the best of our knowledge, this is the first study examining the expression profile of inflammatory lncRNAs in leukocytes of SARS-CoV-2 patients Prior investigations have primarily centered on the analysis of lncRNAs within peripheral blood mononuclear cells (PBMCs) and whole blood. Notably, two of these prior studies utilized in silico methodologies, employing bioinformatics approaches to explore lncRNAs as potential candidates involved in various adverse reactions associated with COVID-19 [13,14]. In contrast, another study conducted a comprehensive transcriptional analysis of lncRNAs using RNA-Seq on PBMC samples from COVID-19 patients, albeit without subsequent qRT-PCR validation [15]. It is intriguing to note that significant differences in the expression levels of lncRNAs AC000120.7 and SENP3-EIF4A1, as observed in our study, were not reported in these prior investigations. Possible contributors to this discrepancy may encompass methodological disparities, including variations in PBMC acquisition techniques [16,17].

Due to the lack of information in the literature about long non-coding RNAs AC000120.7 and SENP3-EIF4A1, we searched a variety of databases, such as NCBI (https://www.ncbi.nlm.nih.gov/. Accessed on: 5 July 2023), the RNA Interactome Database (https://www.rna-society.org/rnainter/. Accessed on: 5 July 2023), RNA Central (https://rnacentral.org/ Accessed on: 5 July 2023; https://diana.e-ce.uth.gr/lncbasev3. Accessed on: 5 July 2023), LNCipedia (https://lncipedia.org/. Accessed on: 5 July 2023), and LncRRIsearch (http://rtools.cbrc.jp/LncRRIsearch/. Accessed on: 5 July 2023), looking for diverse aliases for those long non-coding RNAs. Consequently, through the bioinformatics tool miRNet 2.0 (https://www.mirnet.ca/. Accessed on: 5 July 2023), we created an interaction pathway with the predicted interactions of lncRNAs AC000120.7 (KRIT1) and SENP3-EIF4A1 with diverse microRNAs and genes. Notably, our observations unveiled the presence of a range of genes linked to inflammation and COVID-19, such as IL-6, basigin, and MMP9. This discovery aligns with the extensive exploration by various researchers into the role of cytokines in the pathophysiology of COVID-19 infection [18,19]. Furthermore, a study conducted by Springall and collaborators revealed an intriguing association of IL-6, MMP9, and other cytokines with CD147, a molecule proposed as a potential entry point for the host receptor in COVID-19 [20].

AC000120.7 is an lncRNA that exhibits increased expression in PSD patients compared to NSD patients. Upon investigating its potential functionality, we observed that AC000120.7 overlaps with the sense strand of the gene encoding the KRIT1 protein [21]. However, since this strand is in the sense orientation, it is unlikely to harm mRNA or protein production. Notably, this lncRNA has also been reported in another inflammatory condition, periodontitis, although the specific molecular mechanisms underlying its involvement in periodontitis have yet to be described [22].

SENP3-EIF4A1 is an lncRNA located on chromosome 17’s p-arm at position 13.1 [20]. Previous studies have linked this lncRNA to hepatocellular carcinoma, suggesting its potential as a diagnostic biomarker for this disease [23]. In addition, it has been described that SENP3-EIF4A1 inhibits the activity of miR-195-5p, as a negative correlation has been observed between miR-195-5p and SENP3-EIF4A1 [24]. Interestingly, this interaction can be observed in our interaction pathway image (Figure 1). The significance of its interaction with miR-195-5p in our results stems from the fact that this miRNA negatively regulates the expression of inflammatory factors, such as vascular endothelial growth factor A (VEGFA) [25]. VEGFA contributes to increased vascular permeability during various stages of inflammation [26], and its presence has been associated with the development of neurological symptoms in COVID-19 patients. VEGF also facilitates the recruitment of inflammatory cells and has been identified as a promising therapeutic target for suppressing inflammation during SARS-CoV-2 infection with neurological symptoms [27]. On the other hand, miR-195 has been implicated in macrophage polarization within the cardiovascular system. It inhibits the mediators of the TLR2 inflammatory pathway and affects the recruitment capacity and migration profile of smooth muscle cells [28]. Notably, COVID-19 is associated with cardiovascular manifestations, such as myocardial injury and arrhythmias. Some patients, especially those without the typical symptoms of fever or cough, may present with cardiac symptoms as the initial clinical manifestation of COVID-19. However, the etiology of heart failure in COVID-19 can arise from factors such as hypoxia, cytokine release, volume overload, renal failure, stress, or critical illness. Furthermore, underlying subclinical heart failure may be uncovered or exacerbated by SARS-CoV-2 infection [29]. Another important aspect to consider is the potential impact of SENP3-EIF4A1 blocking the activity of miR-195-5p. Rat models have demonstrated that when miR-195-5p is overexpressed in vitro and in vivo, there is a significant reduction in the production of pro-inflammatory cytokines in pulmonary macrophages in rats with obstructive pulmonary disease [30]. Finally, the impact of SENP3-EIF4A1 in inhibiting miR-195-5p, along with its subsequent effects on the inhibition of inflammatory factors such as VEGFA, the modulation of the TLR2 inflammatory pathway, and the potential attenuation of pro-inflammatory cytokine production in COVID-19, remain areas that require further investigation.

In the context of COVID-19 infection, our results add to the knowledge that has shown lncRNAs to exhibit potential roles in immune evasion, modulation of cytokine storms, and the regulation of both innate and adaptive immune responses [31]. They may contribute to immune evasion by interfering with crucial immune pathways, possibly binding to the viral genome and, thus, affecting viral replication [17,32]. Moreover, certain lncRNAs enhance innate immune activation and inflammatory responses, such as lnc02384, which promotes IFN-γ synthesis. Additionally, lncRNAs like NORAD, RAD51-AS1, GAS5, NEAT1, and MALAT1 appear to regulate cytokine and chemokine expression, contributing to cytokine storms [11]. While these findings offer insights into lncRNAs’ roles in SARS-CoV-2 infection, further research is required to uncover precise mechanisms. Understanding these mechanisms could have vital implications for antiviral therapies and managing immune responses in COVID-19 patients.

This study presents some limitations that need to be acknowledged. Firstly, the sample size in this research was relatively small, which may limit the generalizability of the results. Although we aimed to compensate for this limitation by conducting a comprehensive analysis of a panel of inflammation-related lncRNAs, the small sample size remained a constraint. Secondly, in this pilot study, our primary objective was to investigate the differences in lncRNA expression between these two specific patient groups, but the absence of a control group consisting of healthy individuals was a limitation. The inclusion of healthy controls would have provided valuable baseline data for the expression levels of the studied lncRNAs and enabled a clearer distinction between individuals with SARS-CoV-2 infection and those without. However, we consider that our data are important because this is the first time that the expression of lncRNAs has been compared between two entities where similarities and differences are both recognized.

We hold the belief that our discoveries possess a provocative essence and will act as a catalyst for forthcoming research initiatives. These studies should primarily focus on validating our results across diverse population groups, utilizing a combination of qRT-PCR, RNA-Seq, and/or in situ hybridization techniques. Furthermore, these investigations should delve more profoundly into unraveling the intricate molecular mechanisms governed by these lncRNAs within the context of SARS-CoV-2 infection. Specifically, the study of lncRNAs holds promise for advancing our future understanding of coronaviruses.

## 5. Conclusions

In conclusion, this study sheds light on the potential roles of lncRNAs AC000120.7 and SENP3-EIF4A1 as candidate biomarkers of COVID-19, particularly in the context of ARDS. We observed elevated expression levels of these lncRNAs in SARS-CoV-2-positive individuals, indicating their potential relevance in the pathogenesis of the disease. However, further functional studies are needed to elucidate their precise roles in COVID-19 and ARDS. Additionally, our findings underscore the importance of considering lncRNAs as potential diagnostic and therapeutic targets in respiratory diseases. Furthermore, validating these lncRNAs’ roles and exploring their clinical applications could have significant implications for the management and treatment of COVID-19 and related respiratory conditions.

## Figures and Tables

**Table 1:** Clinical characteristics of patients with upper respiratory infection symptoms who were negative (NSD) or positive (PSD) for SARS-CoV-2 infection according to RT-PCR.

RT-PCR Positive or Negative Cases		NSD			PSD		
General	Female	x	x	x		x	x
	Male				x		
	Age	54	25	29	45	54	23
Symptoms	Sudden onset of symptoms						
	Fever	x		x			
	Cough	x	x	x	x	x	
	Headache	x		x	x	x	x
	Dyspnea	x				x	
	Irritability		x	x		x	x
	Diarrhea		x	x			
	Chest pain		x			x	
	Chills			x		x	
	Odynophagia	x		x			
	Myalgia	x		x	x	x	
	Arthralgia	x		x		x	x
	Malaise	x		x	x	x	
	Rhinorrhea	x		x			x
	Abdominal pain	x		x			
	Anosmia						x
Comorbidities	Hypertension	x					
	Smoking						x
Treatment	Antipyretics			x	x	x	x
Epidemiological history	Contact with influenza or COVID-19 cases in the last 2 weeks		x				x

NSD (negative for SARS-CoV-2 diagnosis) and PSD (positive for SARS-CoV-2 diagnosis).

**Table 2:** Main clinical and demographic data of patients with acute respiratory distress syndrome, either positive or negative for SARS-CoV-2 infection.

	Negative for COVID-19 (*n* = 5)	Positive for COVID-19 (*n* = 10)
Age, years	64 (53.5–67)	64 (52.5–72.7)
Female, *n* (%)	2 (40)	4 (40)
Male, *n* (%)	3 (60)	6 (60)
Invasive mechanical ventilation	3 (60)	3 (30)
Death (%)	2 (40)	2 (20)
Diabetes (%)	1 (20)	3 (30)
Systemic hypertension	3 (60)	5 (50)

Data are expressed as medians with interquartile ranges for quantitative variables, and as frequencies and percentages for qualitative variables.

## Data Availability

All data generated or analyzed during this study are included in this article. Further inquiries can be directed to the corresponding author.
